# Plasticity of NMDA Receptors at Ventral Hippocampal Synapses in the Infralimbic Cortex Regulates Cued Fear

**DOI:** 10.1523/ENEURO.0354-18.2019

**Published:** 2019-03-22

**Authors:** Omar Soler-Cedeño, Orlando Torres-Rodríguez, Faviola Bernard, Lizette Maldonado, Anixa Hernández, James T. Porter

**Affiliations:** 1 Department of Basic Sciences, Ponce Research Institute, Ponce Health Sciences University, Ponce, PR 00732; 2 Department of Biology, University of Puerto Rico-Ponce, Ponce, PR 00734

**Keywords:** fear conditioning, fear extinction, NMDA receptor, prefrontal cortex, PTSD, ventral hippocampus

## Abstract

The medial prefrontal cortex (mPFC) processes contextual information from the hippocampus to generate appropriate fear responses. In rodents, one path for sending contextual information to the mPFC is via the direct projections from the ventral hippocampus (vHC) to the infralimbic cortex (IL). Plasticity in the synaptic communication from the vHC to the IL could contribute to the behavioral changes produced by the acquisition and extinction of conditioned fear. To examine this possibility, we used optogenetic stimulation of vHC synapses in brain slices from trained rats. We found that fear acquisition reduced NMDA receptor (NMDAR) currents at vHC synapses onto IL pyramidal neurons. The depression of NMDAR currents reversed more efficiently after extinction in the conditioning context than extinction in a novel context. Moreover, a cohort of animals that exhibited poor extinction retrieval failed to reverse the plasticity induced by fear conditioning. In addition, ex vivo application of brain-derived neurotrophic factor (BDNF), which is known to simulate extinction in IL, reversed this conditioning-induced plasticity mimicking extinction. Therefore, we have identified a novel mechanism that modulates conditioned fear via changes in NMDAR current at vHC synapses onto IL pyramidal neurons. Disruption of this mechanism could contribute to the abnormal contextual modulation of fear seen in posttraumatic stress disorder (PTSD).

## Visual Abstract

**Figure:**
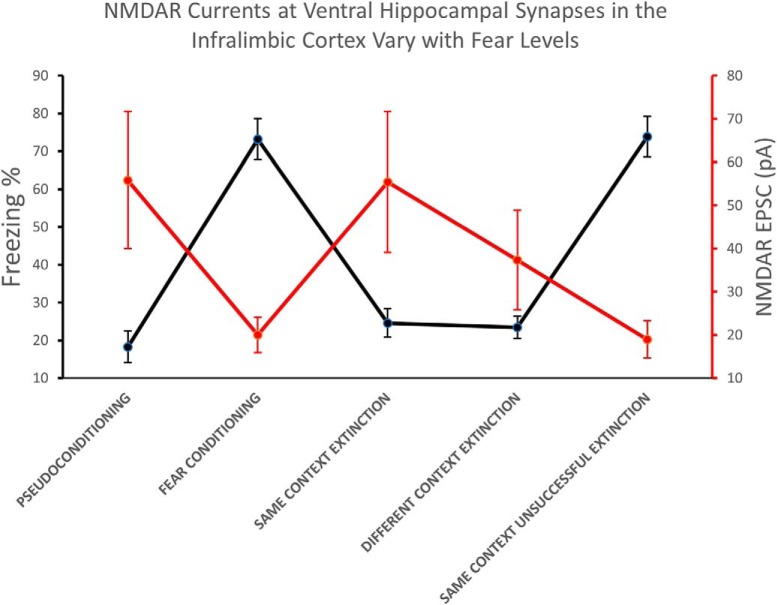

## Significance Statement

Contextual information processing by the medial prefrontal cortex (mPFC) is critical for appropriate behavioral responses. Using optogenetics in brain slices, we found that acquisition of conditioned fear depressed NMDAR currents at ventral hippocampal synapses in the infralimbic cortex (IL) and extinction reversed the depression. Brain-derived neurotrophic factor (BDNF) could also reverse the conditioning-induced depression mimicking extinction. In contrast, animals with impaired fear extinction recall, a posttraumatic stress disorder (PTSD)-like phenotype, failed to reverse the conditioning-induced changes. Our findings suggest that conditioned fear responses are modulated by changes in NMDAR current at ventral hippocampal synapses in IL and suggest a novel mechanism that could contribute to impaired contextual modulation of fear seen in patients with PTSD.

## Introduction

Contextual information is crucial to the generation of appropriate responses to environmental cues. Inappropriate contextual information contributes to various psychopathological conditions. For example, patients with PTSD show deficits in discriminating safe and dangerous contexts (Rougemont-Bücking et al., 2011; Garfinkel et al., 2014). Functional imaging studies show that patients with PTSD have abnormal activity in the ventromedial prefrontal cortex (vmPFC), amygdala, and hippocampus during Pavlovian fear conditioning and extinction (Milad et al., 2009; Rougemont-Bücking et al., 2011). These brain structures are closely implicated in contextual processing (Maren et al., 2013) suggesting that deficits in activation of these structures likely contribute to the discrimination deficits seen in these patients (Garfinkel et al., 2014).

Fear extinction is context-dependent (Bouton and Bolles, 1979; Bouton, 2004; Kalisch et al., 2006; Langton and Richardson, 2009) and involves an interconnected neural circuit composed of the mPFC, hippocampus, and amygdala (Kalisch et al., 2006; Milad et al., 2007). The ventral hippocampus (vHC) conveys contextual information and synaptically excites neurons in the infralimbic cortex (IL); Maren et al., 2013; Kim and Cho, 2017, the homologous region to the vmPFC in rodents. Since the stimulation of IL pyramidal neurons inhibits conditioned fear (Milad et al., 2004; Bukalo et al., 2015; Do-Monte et al., 2015), synaptic communication between the vHC and IL could mediate the context-dependency of extinction (Sierra-Mercado et al., 2011; Orsini et al., 2013; Kim and Cho, 2017; Marek et al., 2018). However, whether vHC synapses in IL are altered by fear conditioning or extinction remains unknown.

In this study, we investigated whether fear conditioning or extinction alter the synaptic efficacy of vHC synapses onto IL pyramidal neurons. Additionally, we examined whether extinction given in the conditioning context induces similar synaptic plasticity as extinction in a novel context. To address these possibilities, we combined whole-cell patch-clamp electrophysiology and optogenetics in brain slices with Pavlovian fear conditioning and extinction to specifically examine vHC synapses onto IL pyramidal neurons. In contrast to previous studies that did not find any plasticity of globally-activated synapses in IL after fear conditioning (Pattwell et al., 2012; Sepulveda-Orengo et al., 2013), we found that fear conditioning induced plasticity in vHC synapses in IL. Fear conditioning increased the ratio of AMPAR to NMDA receptor (NMDAR)-mediated currents by reducing NMDAR currents at the vHC synapses in IL. This postsynaptic plasticity was reversed more efficiently when extinction was conducted in the conditioning context than in a novel context. Furthermore, animals that showed poor extinction memory, a PTSD-like phenotype, failed to reverse the conditioning-induced plasticity, whereas the plasticity could be reversed by the ex vivo application of BDNF, which is known to simulate extinction when infused into IL (Peters et al., 2010; Rosas-Vidal et al., 2014).

## Materials and Methods

### Animal subjects

All animal procedures were approved by the Institutional Animal Care and Use Committee in compliance with NIH guidelines for the care and use of laboratory animals. Male Sprague Dawley rats were transported from the institutional colony to a satellite facility nearby where they were individually housed on a 12/12 h light/dark schedule with free access to food and water.

### Stereotaxic surgery

Rats (between 90 and 120 g of weight) received bilateral injections of an AAV5 vector (1.0 μl) expressing channelrhodopsin-2 (ChR2) and enhanced yellow fluorescent protein (EYFP) driven by the neuron-specific CaMKII promoter (AAV-CaMKIIa-hChR2(H134R)-EYFP; University of North Carolina at Chapel Hill Vector Core Services) into the vHC via a 5-μl Hamilton syringe using stereotactic coordinates (Sierra-Mercado et al., 2011). After waiting a period of 8–12 weeks for recovery and adequate ChR2 expression, rats received behavioral training. After completing behavioral training, animals were euthanized for acute brain slice preparation. In addition, brain sections containing the hippocampus were fixed and prepared for further confirmation of EYFP expression in the vHC.

### Behavioral apparatus

The fear conditioning context (context A) was a chamber of 25 × 29 × 28 cm with aluminum and Plexiglas walls (Coulbourn Inst.). The floor consisted of stainless-steel bars that could be electrified to deliver a mild shock and a single overhead light provided illumination. Context B, which was used to give extinction and recall test to the DIFF group, consisted of a hexagonal chamber with a flat floor, citric scent, and different illumination color. Contexts A and B had speakers mounted on the outside wall and were situated inside a sound-attenuating box (Med Associates) with a ventilating fan that produced an ambient noise level of 60 dB. The conditioned stimulus (CS) was a 4-kHz tone with duration of 30 s and an intensity of 80 dB. The intertone interval for successive tone presentations in the conditioning, extinction, and test phases was an average of 2 min. The unconditioned stimulus (US) was a 0.50-mA scrambled foot shock, 0.5 s in duration, that co-terminated with the tone during the conditioning phase. Behavior was recorded with digital video cameras (Micro Video Products).

### Fear conditioning and extinction

Animals were randomly assigned to one of the following experimental groups: pseudo-conditioned (PSEUDO), fear conditioned (COND), fear extinguished in context A (SAME), or fear extinguished in context B (DIFF). On day 1, animals from the COND, SAME, and DIFF groups received auditory fear conditioning, which consisted of one habituation tone followed by five tone-shock pairings. Animals from the PSEUDO group received the same amount of tones and shocks but in a non-paired manner. On day 2, PSEUDO and COND animals remained in their home cages while SAME and DIFF animals received two sessions of 15 tone-alone presentations, with 1 h between sessions, in context A and context B, respectively. On day 3, rats from the PSEUDO, COND, and SAME groups received two tone-alone presentations in context A, whereas rats from the DIFF group received two tone-alone presentations in context B. For the BDNF experiments, animals received fear conditioning on day 1 and recall test on day 2.

### Whole-cell recordings

Immediately after the test on day 3, rats were deeply anesthetized with pentobarbital (65 mg/kg) and perfused transcardially with ice-cold *N*-methyl-D-glucamine (NMDG) based artificial CSF (ACSF) and decapitated. Brains were quickly removed and placed in ice-cold NMDG ACSF. Then, 300-μm coronal slices of the mPFC were cut with a Vibratome 1000 Plus (Vibratome). We used a modified NMDG-based ACSF to obtain healthy brain slices from adult animals. The composition of the NMDG-based ACSF (Ting et al., 2014) was the following: 93 mM NMDG, 2.5 mM KCl, 1.2 mM NaH_2_PO_4_, 30 mM NaHCO_3_, 20 mM HEPES, 25 mM glucose, 5 mM sodium ascorbate, 2 mM thiourea, 3 mM sodium pyruvate, 10 mM MgSO_4_, and 0.5 mM CaCl_2_. The mPFC slices were initially incubated at 33°C in NMDG ACSF for 12 min before being transferred to an additional 1-h incubation in modified HEPES ACSF at room temperature (21–23°C). The composition of the modified HEPES ACSF was the following: 92 mM NaCl, 2.5 mM KCl, 1.2 mM NaH_2_PO_4_, 30 mM NaHCO_3_, 20 mM HEPES, 25 mM glucose, 5 mM sodium ascorbate, 2 mM thiourea, 3 mM sodium pyruvate, 2 mM MgSO_4_, and 2 mM CaCl_2_. Then, mPFC slices were transferred and submerged in the recording chamber and perfused at 2–3 ml/min with room temperature ACSF with 1 μM tetrodotoxin (TTX) and 100 μM 4-amynopyridine (4-AP) to assess monosynaptic postsynaptic currents. In addition, 10 μM picrotoxin was added to block GABA_A_ postsynaptic currents. The composition of the recording ACSF was the following: 126 mM NaCl, 3 mM KCl, 1.25 mM NaH_2_PO_4_, 1 mM MgSO_4_, 26 mM NaHCO_3_, 20 mM glucose, and 2 mM CaCl_2_ and bubbled with 95% O_2_ and 5% CO_2_. The neurons were visualized with infrared video microscopy using a 40× water-immersion objective on an upright E600FN microscope (Nikon Instruments). Whole-cell recordings were done with glass pipettes with a resistance of 2.5–4 MΩ and filled with cesium gluconate internal solution containing the following: 12 mM TEA-Cl, 140 mM CsOH, 10 mM HEPES, 140 mM gluconic acid, 10 mM biocytin, 2 mM adenosine triphosphate, 3 mM guanosine triphosphate, and 0.4 mM cesium-ethylene glycol-bis(2-aminoethylether)-N,N,N’,N’-tetra acetic acid (Cs-EGTA, 0.4); pH was adjusted to 7.3 with CsOH (300 mOsm). After establishing a whole-cell voltage-clamp recording, the membrane resistance, membrane capacitance, and access resistance were measured. Recordings were filtered at 4 kHz, digitized at 10 kHz, and saved to a computer using pCLAMP9 (Molecular Devices).

### AMPA and NMDAR currents

AMPA and NMDAR-mediated EPSCs in mPFC neurons were measured in response to the optical stimulation of ChR2-expressing vHC axons innervating Layer II/III and V with a 470-nm light-emitting diode (LED; M470L2, Thorlabs) through the 40× objective centered at the soma of the patched neuron with light intensity of 1.5 mW. A total of 106 neurons were recorded from IL (89% in Layer V; 11% in Layer II/III). Picrotoxin was added to the bath to block GABA_A_-mediated currents. TTX and 4-AP were added to ensure monosynaptic measurements. To evoke synaptic responses in the mPFC by photostimulation of vHC fibers, we illuminated slices every 10 s with light pulses of 5- to 20-ms duration. AMPAR-mediated EPSCs were measured as the peak of the EPSCs recorded at –70 mV. NMDAR-mediated EPSCs were measured as the amplitude of the EPSC at +40 mV, 70 ms after the light stimulus. Initial experiments with NMDAR blockers showed that EPSCs contained minimal contamination with AMPAR EPSCs at this time point. Measurements of AMPAR and NMDAR EPSCs were also taken at 0 mV to calculate ion conductance from the current (I) versus voltage (V) plot and peak values were taken similarly to at –70 and +40 mV. AMPAR conductance values were calculated by taking the slope of the I/V plot of AMPA EPSCs measured at holding potentials of –70 and 0 mV, whereas NMDAR conductance values were calculated from the I/V plot values of NMDAR EPSCs measured at holding potentials of 0 and +40 mV.

### BDNF experiments

We prepared mPFC slices from rats that received fear conditioned on day 1 and a fear recall test on day 2. The slices underwent the same recovery process as described above in the whole-cell recordings section. After the slice recovery period, mPFC slice hemispheres were separated and one hemisphere was randomly selected for an additional 1-h incubation in room temperature HEPES-ACSF containing 2 nM BDNF (Invitrogen). The other hemisphere was incubated an additional hour in control HEPES-ACSF. After the 1-h incubation with BDNF or control HEPES-ACSF, slices were transferred to the recording chamber and AMPAR- and NMDAR-mediated EPSCs evoked by optical stimulation of vHC axons were recorded in IL pyramidal neurons.

### Histology

In all experiments, 10 mM biocytin was included in the recording solution to label the neurons for *post hoc* morphologic identification of IL pyramidal neurons. After completing electrophysiological recordings, the slices were fixed overnight in 4% paraformaldehyde. Recorded IL neurons were stained with a standard avidin-biotin peroxidase procedure (Vectastain ABC kit; Vector Laboratories) and visualized with bright-field microscopy. Neurons that were not located in the IL or that were not pyramidal-shaped with obvious apical dendrites were excluded from the analysis.

### Statistical analysis

Freezing behavior was used as an indicator of fear and was assessed uniformly using computer-based analysis program (FreezeScan, Clever Systems). All behavioral data were compared with repeated measures ANOVA followed by Tukey HSD *post hoc* test (IBM SPSS Statistics, IBM Corp.). The electrophysiological data were analyzed using Clampfit (Molecular Devices) and were compared with non-parametric Kruskal–Wallis test or Mann–Whitney test due to skewness of the data in its distribution (IBM SPSS Statistics, IBM Corp.). Significant main effect with Kruskal–Wallis test was followed by Dunn’s *post hoc* test. Effect sizes between means were assessed using Cohen’s *d* (Cohen, 1988; Lenhard and Lenhard, 2016). Values are reported as the mean ± SEM. All animals in which we were able to obtain stable optically-evoked EPSCs were included in the behavioral analysis. Since we wanted to correlate synaptic changes with behavioral changes, we analyzed rats from the extinction groups that showed <50% freezing during the extinction recall tones on day 3 (successful extinction) separate from those rats that failed to recall extinction (unsuccessful) and froze >50% during the recall tones on day 3. Seven of the 13 rats that were extinguished in the fear conditioning context showed successful extinction recall in the conditioning context. All seven rats that received extinction in the novel context showed successful extinction recall in the novel context.

## Results

**Figure 1.**
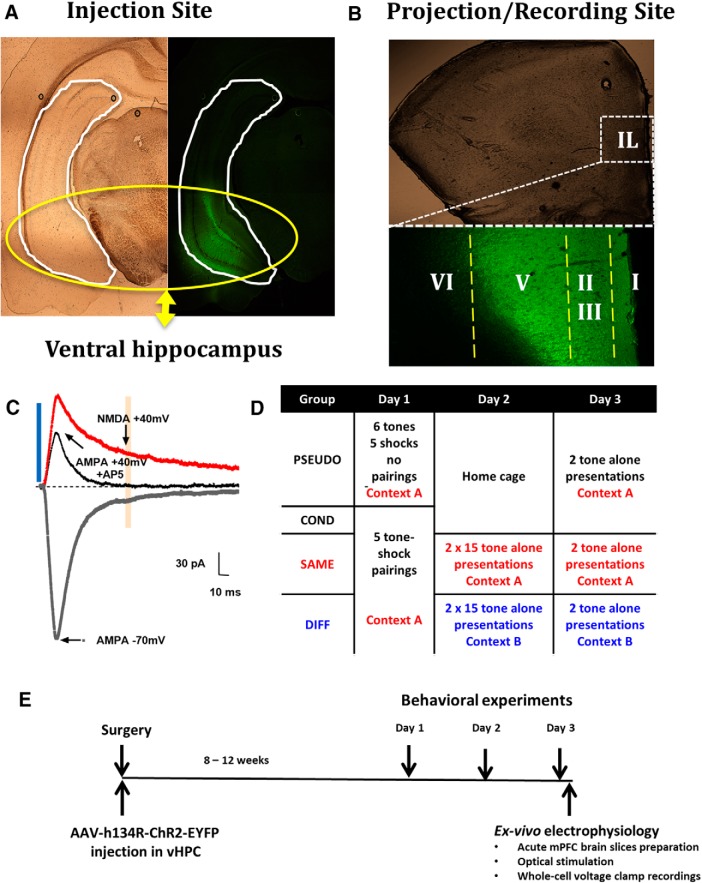
Experimental design to measure vHC EPSCs in IL neurons after fear conditioning and extinction. ***A***, ***B***, ChR2 expression in the vHC and mPFC. ***C***, AMPAR-mediated EPSCs were measured at the peak of the –70-mV current. NMDAR-mediated EPSCs were measured 70 ms after stimulation to minimize the possibility of contamination with AMPAR EPSC. ***D***, Behavioral groups. ***E***, Scheme of the general timeline for the experiments.

**Figure 2.**
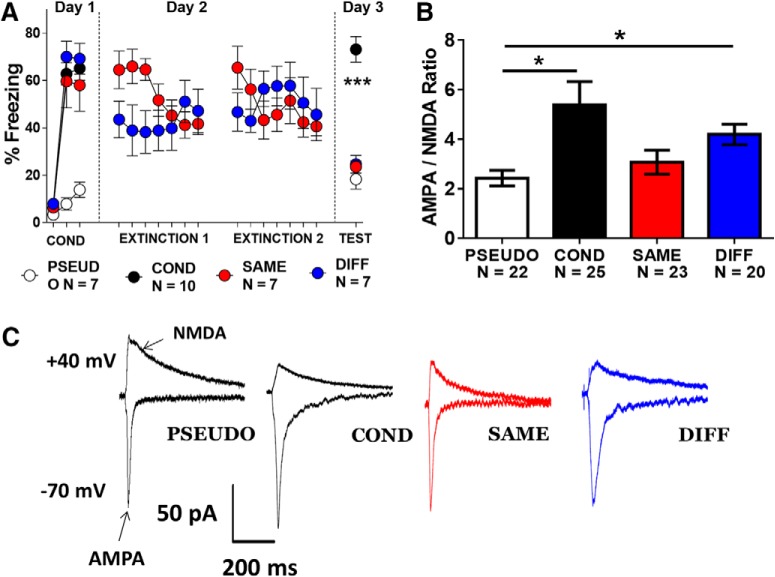
Fear conditioning and extinction induce postsynaptic plasticity in vHC synapses onto IL pyramidal neurons. ***A***, Behavioral responses of all groups. ***B***, Mean AMPA/NMDA ratios measured in IL pyramidal neurons from each group. ***C***, Example traces of vHC EPSCs recorded in IL pyramidal neurons of each group (***A***, ***p < 0.001, repeated measures ANOVA, COND vs PSEUDO, SAME, and DIFF; ***B***, *p < 0.05, Dunn’s *post hoc* after significant Kruskal–Wallis test).

First, AAV vectors expressing ChR2 and EYFP were injected into the vHC of rats. Two to three months later, robust expression of the viral proteins could be seen in the vHC and in the axons in the mPFC (Fig. 1*A*,*B*). Optical stimulation of ChR2-expressing vHC axons evoked EPSCs in IL pyramidal neurons. Whole-cell recordings at –70 mV allowed the measurement of AMPA receptor-mediated EPSCs from the peak current (Fig. 1*C*). Optical stimulation at +40 mV evoked both AMPA and NMDAR currents. Initial experiments with NMDAR blockers showed that 70 ms after stimulation the NMDAR EPSCs contained minimal contamination with AMPAR EPSCs (Fig. 1*C*). Next, we designed four experimental groups to test whether fear conditioning or extinction induces changes in vHC synapses in IL, and whether extinction in the same context (SAME) will induce different synaptic changes in vHC synapses than extinction in a novel context (DIFF; Fig. 1*D*). On day 1, the group that received conditioning but no extinction (COND, *n* = 10) and the SAME (*n* = 7) and DIFF (*n* = 7) groups acquired similar levels of fear after receiving auditory fear conditioning in context A (Fig. 2*A*). The PSEUDO (*n* = 7) group received unpaired tone and shock presentations in context A to avoid association of the tone with the shock. Then, on days 2 and 3, the SAME group received fear extinction and extinction retrieval test in context A whereas the DIFF group received extinction and retrieval test in context B. On day 3, we tested all animals for cued fear and euthanized animals immediately after the test. A cohort of rats in the SAME group were removed from the analysis due to poor extinction memory recall on day 3 and were analyzed separately in Figure 4. As expected (Fig. 2*A*), a repeated measures ANOVA showed a significant main effect (*F*_(2,12)_ = 40.63, *p* < 0.001) and *post hoc* analysis confirmed that rats from the COND group had significantly higher levels of freezing to the tone on day 3 compared to rats from the SAME, DIFF, and PSEUDO groups (*p* < 0.001). The difference in fear expression among groups indicates that all animals were successful in learning their respective behaviors. It is important to point out that the DIFF group received extinction and extinction recall in a novel context. Therefore, the DIFF group did not receive extinction of the conditioning context. Given the abundant evidence of fear renewal (Bouton et al., 2006), the DIFF group would likely show higher freezing in the conditioning context.

### Fear conditioning and extinction induce plasticity in vHC-IL synapses

After recall on day 3, we prepared acute brain slices and used whole-cell voltage-clamp recordings to assess AMPA to NMDA ratios in mPFC pyramidal neurons after selective optical stimulation of vHC axons (Fig. 2*B*,*C*). The following number of cells was evaluated in each group: PSEUDO (*n* = 22 cells from seven rats), COND (*n* = 25 cells from 10 rats), SAME (*n* = 23 cells from seven rats), and DIFF (*n* = 20 cells from seven rats). Kruskal–Wallis test showed a significant main effect in AMPA to NMDA ratios of vHC inputs in IL (*H*_(3,90)_ = 14.70, *p* = 0.002). Dunn’s *post hoc* analysis revealed a significant increase in AMPA to NMDA ratios in the COND (*p* = 0.012) and DIFF (*p* = 0.018) groups compared to the PSEUDO group but not the SAME group (Fig. 2*B*; PSEUDO vs SAME, *p* = 1.00; COND vs SAME, *p* = 0.12; COND vs DIFF, *p* = 1.00; SAME vs DIFF, *p* = 0.15). Moreover, large effect sizes in AMPA to NMDA ratios were found between the COND (*d* = 0.8) and the DIFF (*d* = 1.1) groups compared to the PSEUDO group. SAME versus COND (*d* = 0.6) and SAME versus DIFF (*d* = 0.5) showed intermediate effect sizes. Small effect sizes were found between PSEUDO versus SAME (*d* = 0.3) and COND versus DIFF (*d* = 0.3). Therefore, a significant reversal of the AMPA to NMDA ratio increase found in the COND group was observed only in the SAME group. These findings suggest that fear conditioning induces postsynaptic plasticity of vHC inputs in IL that is more effectively reversed when extinction occurs in the same context as conditioning.

### Fear conditioning and extinction alter NMDAR currents in vHC-IL synapses

**Figure 3.**
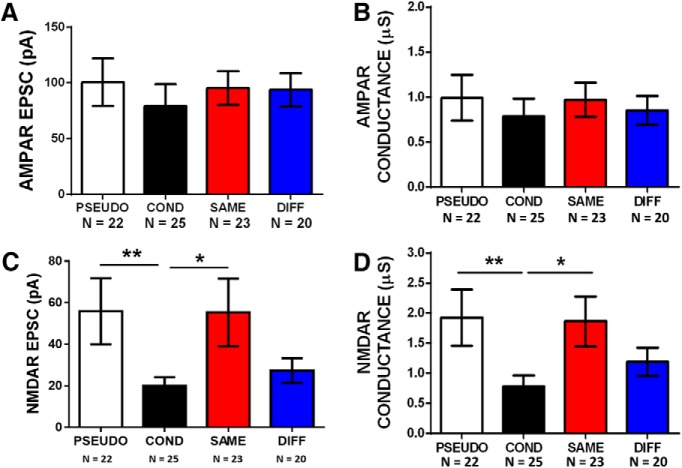
Fear conditioning and extinction induced bidirectional changes in NMDAR currents at vHC synapses onto IL pyramidal neurons. ***A***, ***B***, Average AMPAR-mediated EPSCs and conductance in each group. ***C***, ***D***, Average NMDAR-mediated EPSCs and conductance in each group (**p < 0.01, *p < 0.05; Dunn’s *post hoc* after significant Kruskal–Wallis test).

Since changes in either AMPAR or NMDAR-mediated EPSCs could produce a change in the AMPA to NMDA ratios, we evaluated the AMPAR and NMDAR components separately to determine which one was responsible for the changes observed in IL neurons (Fig. 3). First we examined the AMPA component and found no significant differences in AMPAR EPSCs (*H*_(3,90)_ = 5.48, *p* = 0.14) or conductance (*H*_(3,90)_ = 1.95, *p* = 0.58; Fig. 3*A*,*B*). In contrast, we found that fear conditioning significantly reduced NMDAR EPSCs and extinction reversed the reduction (Fig. 3*C*). Kruskal–Wallis test showed a significant main effect in NMDAR EPSCs (*H*_(3,90)_ = 15.98, *p* = 0.001) and *post hoc* analysis revealed that NMDAR EPSCs were smaller in the COND group compared to PSEUDO (*p* = 0.002) and SAME (*p* = 0.023) groups (COND vs DIFF, *p* = 1.00; PSEUDO vs SAME, *p* = 1.00; PSEUDO vs DIFF, *p* = 0.12; SAME vs DIFF, *p* = 0.65). Cohen’s *d* found intermediate effect sizes in NMDAR EPSCs in PSEUDO versus COND (*d* = 0.7), PSEUDO versus DIFF (*d* = 0.5), COND versus SAME (*d* = 0.6) and SAME versus DIFF (*d* = 0.5). Small effect sizes were found in COND versus DIFF (*d* = 0.3), and no effect in PSEUDO versus SAME (*d* = 0.01). In addition, we found that fear conditioning decreased NMDAR conductance and extinction reversed the decrease in NMDAR conductance (Fig. 3*D*). Kruskal–Wallis test showed a significant main effect in NMDAR conductance (*H*_(3,90)_ = 14.26, *p* = 0.003) and *post hoc* analysis revealed that NMDAR conductance was smaller in the COND group compared to PSEUDO (*p* = 0.003) and SAME (*p* = 0.023) groups (COND vs DIFF, *p* = 0.53; PSEUDO vs SAME, *p* = 1.00; PSEUDO vs DIFF, *p* = 0.60; SAME vs DIFF, *p* = 1.00). Cohen’s *d* found intermediate effect sizes in NMDAR conductance in COND versus PSEUDO (*d* = 0.7) and COND versus SAME (*d* = 0.7). Small effect sizes were found in PSEUDO versus DIFF (*d* = 0.4), COND versus DIFF (*d* = 0.4) and SAME versus DIFF (*d* = 0.4). These results suggest that the changes observed in AMPA to NMDA ratios were caused by alterations in NMDAR mediated currents induced by fear conditioning and extinction. Acquisition of fear reduced the NMDAR-mediated currents at vHC synapses onto IL neurons and extinction in the same context reversed this depression. Extinction in a novel context failed to reverse the depressed NMDAR currents.

### Failure to recall extinction correlates with failure to reverse conditioning-induced depression of NMDAR currents in vHC-IL synapses

**Figure 4.**
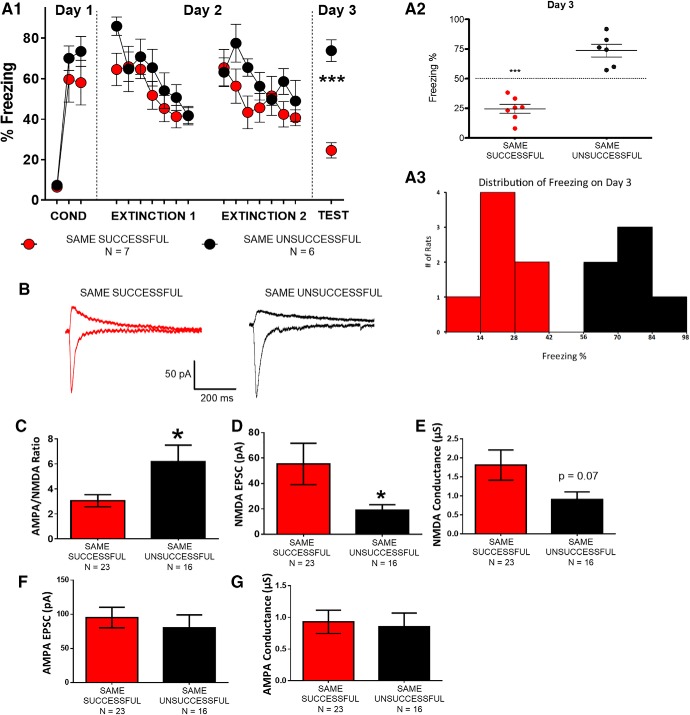
IL neurons from animals that failed to recall extinction showed greater AMPA/NMDA ratios and reduced NMDAR-mediated currents. ***A***, Behavioral data from same context extinction animals that showed successful or failed extinction recall. ***B***, Example vHC EPSCs recorded in IL pyramidal neurons. ***C***, Average AMPA/NMDA ratio in each group. ***D***, ***E***, Average NMDAR-mediated EPSC and conductance in each group. ***F***, ***G***, Average AMPAR-mediated EPSC and conductance in each group (*p < 0.05; Mann–Whitney’s test).

As found previously by others (Burgos-Robles et al., 2007; Peters et al., 2010; Gruene et al., 2015), we found a cohort of animals that received extinction in the fear conditioning context (SAME, from Fig. 2*A*) that showed poor extinction memory retrieval (UNSUCCESSFULL; Fig. 4*A*). Thus, although these animals received the same behavioral treatment as the SAME group in Figure 2*A*, they showed poor extinction memory with >50% freezing during the extinction recall test (UNSUCCESSFUL group; Fig. 4*A*). In fact, the UNSUCCESSFULL group behaved as though it never received extinction and froze as much as the COND group at recall (*t* = 0.07634, df = 14, *p* = 0.9402). In comparison, the SAME group from Figure 2*A* showed <50% freezing during recall (SUCCESSFUL group; Fig. 4*A*). If the reversal of NMDAR currents by extinction is important for extinction memory, then animals that failed to remember extinction should show smaller NMDAR currents. Consistent with this, AMPA to NMDA ratios of vHC inputs in IL of the UNSUCCESSFUL (*n* = 16 cells from six rats) group were greater than those from rats with successful extinction retrieval (SUCCESSFUL, *n* = 23 cells from seven rats; Mann–Whitney *U* test, *U* = 108, *p* = 0.030). In addition, a large effect size was found in AMPA to NMDA ratios between the groups (*d* = 0.8). As observed in COND animals (Fig. 3*D*), the larger AMPA to NMDA ratios in the UNSUCCESSFUL group was caused by smaller NMDAR EPSCs (Fig. 4*D*,*E*). Mann–Whitney *U* test showed that the UNSUCCESSFUL group had smaller NMDAR EPSCs (*U* = 266, *p* = 0.019) with an intermediate effect size (*d* = 0.6). Also, NMDAR conductance in UNSUCCESSFUL animals showed a tendency toward a reduction (*U* = 248, *p* = 0.069) with an intermediate effect size (*d* = 0.6). Once again, no differences were found in the AMPAR current (*U* = 216, *p* = 0.37) or conductance (*U* = 192, *p* = 0.83; Fig. 4*F*,*G*). These findings suggest that the observed changes in NMDAR currents at vHC synapses in IL are important for successful extinction memory retrieval.

**Figure 5.**
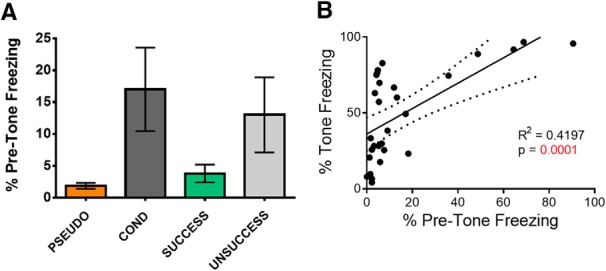
Contextual fear to context A varies with fear to tones. ***A***, Average freezing during the minute before giving the first test tone on day 3 in the different groups. ***B***, Correlation between freezing before the tones and during the tones on day 3. Rats from the PSEUDO, COND, SUCCESSFUL, and UNSUCCESSFUL groups are plotted.

During auditory fear conditioning, the rats learn to associate the tone and context with the aversive foot shock. Therefore, differences in contextual fear among the behavioral groups may also contribute to the synaptic differences. To address this issue, we calculated the percentage of time the rats froze during the minute before the first tone on day 3 which is a measure of fear to the context A for the PSEUDO (*n* = 7), COND (*n* = 10), SUCCESSFUL (*n* = 7), and UNSUCCESSFUL (*n* = 6) groups. The DIFF group that received extinction in a novel context B were not included since their recall was given in context B and not in the fear conditioning context A. Although the contextual fear varied among the groups (Fig. 5*A*), there were no significant differences among them (*F*_(3,25)_ = 2.394, *p* = 0.092). However, the small sample size likely affected this statistical analysis; therefore, we also calculated effect size. When we evaluated the effect size, we found a large overall effect in contextual freezing among groups (*d* = 1.0). A large effect in contextual freezing was observed in the COND group compared to the PSEUDO (*d* = 0.9) and SAME (*d* = 0.8) groups. An intermediate effect in contextual freezing between the PSEUDO (*d* = 0.6) and SAME (*d* = 0.5) groups compared to the UNSUCCESFUL group. A small effect between the COND group compared to the UNSUCCESSFUL group (*d* = 0.2) and no effect between the PSEUDO and SAME groups (*d* = 0.1). Therefore, as expected the COND and UNSUCCESSFUL groups showed more contextual fear than the PSEUDO and SAME groups. Furthermore, the contextual fear shown on day 3 strongly correlated with the cued fear to the tones (Fig. 5*B*).

### BDNF mimicked fear extinction by reversing fear conditioning-induced increase in AMPA/NMDA ratios and decrease in NMDAR currents in vHC-IL synapses

**Figure 6.**
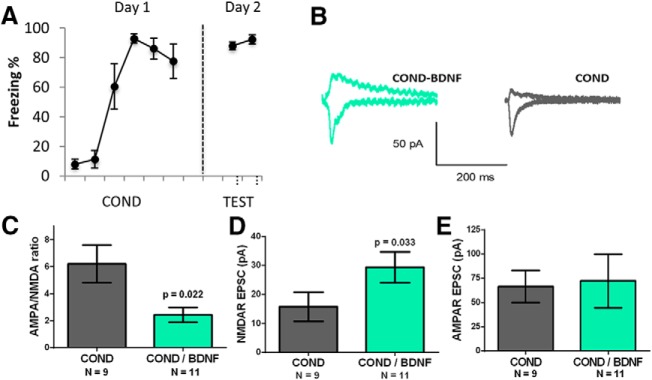
*In vitro* BDNF treatment mimicked fear extinction reversal of conditioning-induced changes in AMPA/NMDA ratio and NMDAR currents. ***A***, Behavioral data. ***B***, Example trace for each group. ***C***, Average AMPA/NMDA ratio for each group. ***D***, Average NMDAR EPSC for each group. ***E***, Average AMPAR EPSCs (*p* < 0.05, Mann–Whitney’s test).

Previous studies have nicely demonstrated that *in vivo* infusion of BDNF into IL before extinction is sufficient to decrease fear expression and induce extinction (Peters et al., 2010; Rosas-Vidal et al., 2014). Therefore, if the changes we observed in the NMDAR currents are important for extinction, then BDNF should produce similar changes in the NMDAR currents at the vHC to IL synapses as fear extinction. As shown in Figure 6, we found that BDNF-treated IL neurons (*n* = 11 cells from four rats) from fear conditioned animals showed lower AMPA/NMDA ratios and larger NMDAR EPSCs than non-treated IL neurons (*n* = 9 cells from four rats) from the same animals. Mann–Whitney *U* test showed a significant mean difference in AMPA/NMDA ratios (*p* = 0.022) and NMDAR EPSCs (*p* = 0.033). Cohen’s *d* found large effects in AMPA/NMDA ratios (*d* = 1.2) and NMDAR EPSCs (*d* = 0.8) between groups. Thus, ex vivo incubation with BDNF decreased the AMPA/NMDA ratio and increased the NMDAR currents in vHC-IL synapses mimicking the synaptic effects produced by fear extinction. These findings further substantiate that the observed changes in NMDAR currents at vHC-IL synapses contribute to the behavioral changes.

## Discussion

By using optogenetic stimulation, we found that auditory fear conditioning induces postsynaptic plasticity at vHC synapses onto IL pyramidal neurons that involves an increase in the ratio of AMPA to NMDAR currents. Further examination revealed that fear acquisition reduced the NMDAR currents without altering the AMPA currents. Previous studies using electrical stimulation of all axons within the vicinity of the electrode did not observe synaptic plasticity after fear conditioning in IL (Pattwell et al., 2012; Sepulveda-Orengo et al., 2013). This suggests that the depression of NMDAR currents at vHC-IL synapses does not occur at the majority of synapses onto IL neurons and could be a unique feature of vHC synapses. Although we used established coordinates for infusion of the viral constructs into the rat vHC (Sierra-Mercado et al., 2011) and we confirmed expression of viral proteins in the vHC (Fig. 1*A*), it is not possible to completely rule out the possibility that some fibers from viral expression in areas near the vHC also contribute to the inputs recorded in IL. However, based on the relatively robust projections from the vHC to IL (Hoover and Vertes, 2007), the majority of inputs activated in IL likely come from the vHC.

Exposure to extinction reversed the fear conditioning-induced plasticity leading to an increase in NMDAR currents and a corresponding decrease in AMPA/NMDA ratio. In support of our findings, a recent study in mice also found increased NMDA currents at vHC-IL synapses after fear extinction (Wang et al., 2018). In our experiments, extinction induced a more complete reversal of conditioning-induced plasticity when the extinction was conducted in the fear conditioning context. The fact that the group that received cued fear extinction in the novel context showed intermediary changes in the NMDAR EPSCs compared to the group that received extinction in the same context as fear conditioning suggests that extinction of fear to the cue and extinction of fear to the context both contribute to the synaptic modifications of the vHC-IL synapses. Exposure to extinction in the novel context likely left remnants of the contextual fear which produced NMDAR EPSCs in between those of the COND group and the group that received extinction in the fear conditioning context. This suggests that contextual and cued fear modulate vHC-IL synapses independently. Whether the same or independent populations of ventral hippocampal neurons are modulated remains to be determined.

The observed changes in AMPA/NMDA ratio, NMDAR EPSC, and NMDAR conductance at the vHC-IL synapse varied with the levels of fear expression suggesting that these changes contribute to the behavioral outcome. The fact that animals that failed to recall extinction memory did not show a reversal of the conditioning-induced changes suggests that the changes in NMDAR requires the behavioral change rather than simple exposure to extinction training. Moreover, the extinction-induced changes were mimicked by incubating slices with BDNF. Therefore, plasticity of NMDAR currents at vHC to IL synapses appears to modulate cued fear expression and represents a novel mechanism for modulating conditioned fear.

Our findings suggest that learning-induced changes in NMDAR currents occur in response to the acquisition and extinction of conditioned fear. Consistent with our findings, physiologically relevant stimuli in brain slices can induce selective changes in NMDAR currents without altering AMPA receptor currents (Kwon and Castillo, 2008; Rebola et al., 2008). Furthermore, NMDAR show bidirectional plasticity in response to synaptic stimulation in slices (Rebola et al., 2010; Hunt and Castillo, 2012). Similar to our findings, fear learning decreased NMDAR currents in the amygdala by reduced phosphorylation of the GluN1 subunit of NMDAR (Zinebi et al., 2003). Although acute stress can depress NMDAR currents (Kuzmiski et al., 2010), it is unlikely that the changes in NMDAR currents we observed were mediated by stress, since our results were compared to the pseudoconditioned group that should have similar stress exposure.

Previous studies demonstrated that infusion of BDNF into IL is sufficient to simulate extinction and reduce fear to a CS (Peters et al., 2010; Rosas-Vidal et al., 2014). This effect of BDNF was prevented by systemic blockade of NMDARs (Peters et al., 2010) suggesting that BDNF may increase NMDA currents to reduce conditioned fear. Consistent with this possibility, we found that ex vivo incubation of prefrontal slices with BDNF induced similar synaptic changes in the vHC inputs in IL as extinction. Another recent study in mice also showed that incubation with BDNF increases the NMDA currents at vHC synapses in IL (Wang et al., 2018). Therefore, one synaptic mechanism by which BDNF simulates extinction may be by increasing NMDAR currents at vHC inputs in IL pyramidal neurons. However, an infusion of BDNF *in vivo* may also induce additional cellular and circuit effects that contribute to the reduction in conditioned fear.

Inputs from vHC to the mPFC provide spatial representations that guide aversive behaviors (Padilla-Coreano et al., 2016). Our findings suggest that the behavioral outcome of vHC stimulation of IL is altered by fear conditioning and extinction. Consistent with IL’s proposed role of inhibiting freezing in response to aversive cues (Milad and Quirk, 2002; Sierra-Mercado et al., 2011; Moscarello and LeDoux, 2013), weakened vHC activation of IL pyramidal neurons after fear conditioning would increase freezing behavior. This reduced synaptic activation of IL excitatory neurons combined with the strong feedforward inhibition produced by vHC inputs in IL (Marek et al., 2018) and depressed intrinsic excitability of IL pyramidal neurons after fear conditioning (Santini et al., 2008; Soler-Cedeño et al., 2016) would reduce IL activation of downstream targets such as the amygdala (Quirk et al., 2003; Cho et al., 2013) and enhance acquisition of conditioned fear. In support of this model, stimulation of IL excitatory neurons impairs the acquisition of conditioned fear (Yizhar et al., 2011) as does increased glucocorticoid signaling in IL (Criado-Marrero et al., 2017).

After fear extinction, the increase in vHC activation of NMDAR currents on IL neurons would increase the activation of IL pyramidal neurons (Orsini et al., 2011; Kim and Cho, 2017). The slower kinetics of NMDARs allow them to drive neuronal burst firing (Polsky et al., 2009; Grienberger et al., 2014). Therefore, the relative increase in NMDAR currents after extinction in the conditioning context likely contributes to the increase in NMDAR-dependent burst firing seen in IL neurons shortly after acquisition of fear extinction which correlated with good extinction memory (Burgos-Robles et al., 2007). The resulting increased activation of the amygdala by IL projections would produce a context-dependent modulation of conditioned fear (Orsini et al., 2011; Maren et al., 2013).

In conclusion, we have found that fear conditioning and extinction induce bidirectional changes in NMDAR currents at vHC synapses in IL. Acquisition of conditioned fear reduced NMDAR currents, while extinction enhanced the NMDAR currents. Failure to reverse the conditioning-induced depression of NMDAR currents led to poor extinction memory and a PTSD-like phenotype. Medications designed to activate BDNF receptors may be useful for enhancing NMDAR currents at vHC synapses in IL and treating PTSD.

## Synthesis

Reviewing Editor: Cheryl Sisk, Michigan State University Decisions are customarily a result of the Reviewing Editor and the peer reviewers coming together and discussing their recommendations until a consensus is reached. When revisions are invited, a fact-based synthesis statement explaining their decision and outlining what is needed to prepare a revision will be listed below. The following reviewer(s) agreed to reveal their identity: Alfred Robison, Pedro Bekinschtein.

Reviewers agreed that findings from these experiments are interesting and timely in investigating vHC-IL connections and synaptic plasticity in the context of fear conditioning and extinction. However, there were substantial concerns about some of the statistical analyses, interpretations, and conclusions. It is essential that you provide a rationale for the statistical analyses employed and report sample sizes for all groups and exact p values. Please also address each of the following points in a revised manuscript.

Major Concerns:

1. Full statistics would be more useful to understand the results. The number of subjects or cells should be stated for each experiment in the main text or in the figure captions. Exact p values and effect sizes should be reported.

2. The representative expression of the vector in Fig 1A shows AAV-mediated YFP expression in the hippocampus, however there is also considerable expression in cortex & amygdala transition area, which includes posterior basolateral amygdala, posterior piriform, and entorhinal cortex. The authors cannot rule out that the plasticity is primarily occurring at synapses from neurons from these areas projecting to the IL. This makes it difficult to support the authors' interpretation that vHPC-IL circuits specifically have the plasticity phenotype studied. Further experiments may be required to demonstrate whether these regions project to IL. Alternatively, authors need to adjust their interpretation to include other potential inputs to IL.

3. The authors report correlations as an indicator of a relationship between the freezing behavior and NMDAR plasticity, therefore implying that the plasticity may underlie behavioral changes. However, the authors attempt to make conclusions from improperly performed correlations across all groups. The authors have already demonstrated that NMDAR currents are significantly different between groups, which matches the same pattern that they observe in the behavior, thus the correlation is likely due to group differences and not attributed to individual variation. In order to demonstrate individual variation, and thus a clear relationship between the behavior and plasticity, the authors need to conduct correlations within groups. A significant correlation within the COND group that is not present in the PSEUDO group, and reversed in the SAME (and/or DIFF) groups would be required to make the conclusion that the plasticity and behavior are causally related.

4. Discussion of how individual variation (“PTSD-like behavior”) in extinction predicts NMDAR plasticity is difficult to understand and may be a misrepresentation of the actual findings. The authors have not demonstrated that the individual variation predicts susceptibility to PTSD-like behavior (see #3 above) and need to avoid making broad assumptions about their current data. Discussion of how the individual variation in behavior, which predicts differences in NMDAR current & conductance, is similar to PTSD should therefore be relegated to the discussion only, and PTSD language should be removed from the results section.

5. The discussion of successful vs unsusccessful needs to be described in more detail in the Results section (and potentially Methods). Were the rats in the SAME context cohort in 2A treated with the same protocol as the rats in 4A, as the paper implies? If so, why were there no “unsuccessful” rats in 2A? What is the average spread of freezing across the same context cohort? Was the cohort in Fig 4A an anomaly? In the experiment shown in figure 4, the animals were divided into successful and unsuccessful extinction using a cutoff of 50% time freezing. However, this seems arbitrary. Is this always a population cut-off (pre-set value) or does this change based on the cohort? How is unsuccessful extinction defined? In many studies, animals show freezing responses as lower as 30%, and those are considered significant memory retention scores. This is a very important point since the results really depend on how these groups were constructed. The authors should consider analyzing their data on AMPA/NMDA ratio and conductance using different retention scores cutoffs to separate successful and unsuccessful extinction. This parametrical analysis would be useful to identify exactly at which % freezing the differences in plasticity are evident. If the authors actually did this, then they should report it.

6. One of the main conclusions from experiments depicted in figures 2 and 3 is that the AMPA/NMDA ratio is reversed after extinction. However, the data points at something perhaps more subtle that is yet to be identified. The concern comes from the results observed in the DIFF group, which extinguished at the same level than the SAME group, but does not show significant differences in AMPA/NMDA ratio or NMDA EPSC with respect to the COND group. However, these differences are also not significant compared to the SAME or PSEUDO groups. It would be useful to see the exact p values for all the comparisons and the effect sizes. Also, could the authors test the validity of the null hypothesis? We believe there could be more information to gather from these experiments, simply claiming, “extinction in a novel context failed to reverse the depressed NMDAR currents” is misleading, as the results do not seem to point in that direction.

7. The bar chart in figure 5A does not reflect what is stated in the main text. Again, we understand that the statistical analysis the authors did not detect a significant difference, but simply stating, “Although the contextual fear varied among the groups (Fig. 5A), there were no significant differences among them (F (3, 25) = 2.394, p = 0.092) does not reflect the nature of the results. In fact, the p value is close to 0.05 and we assume that effect sizes are large. This could be resolved by increasing the number of subjects or assays; however, there is no mention of the number of subjects or cells nearly anywhere in the manuscript.

8. Although we understand the logic for doing experiment 7, we believe the comparison with the study done by Peters et al. is limited. The authors conclude, ”BDNF decreased the AMPA/NMDA ratio and increased the NMDAR currents in vHC-IL synapses mimicking the effects produced by fear extinction“. Since BDNF was applied to the bath, it is not possible to associate this mechanism with the one described in the previous experiments. A more comparable experiment would consist on BDNF injection into IL before extinction and slice preparation from these animals. In this case, they could compare memory retention scores with AMPA/NMDA ratios based on the effects of BDNF. In addition, it is unclear if the plasticity reversed by BDNF is acutely disrupted, leading to extinction effect. Additional experiments or appropriate justification may be needed to understand the molecular mechanism for BDNF-mediated changes in extinction and plasticity.

9. Discussion, lines 339-343: ”the fact that the group that received cued fear extinction in the novel context showed intermediary changes in the NMDAR EPSCs compared to the group that received extinction in the same context as fear conditioning suggests that extinction of fear to the cue and extinction of fear to the context both contribute to the synaptic modifications of the vHC-IL synapses.“ We wonder whether this can be concluded from these results or more experiments are required. There is an interesting correlation analysis done in figure 6, maybe it would be worth establishing whether AMPA/NMDA ratio correlates with freezing behavior in the DIFF group; this could give additional information to what is going on there.

10. In lines 369 to 373, the authors discussed their BDNF result. We found this discussion unsatisfactory for the reasons mentioned above. The authors could mention the limitations of this study in comparison with Peters et al. instead of just stating that BDNF may act through activation of Fyn kinase, which sounds a bit incomplete and random, as many other cascades could be involved. No discussion or justification for mention of Fyn kinase is given.

Minor points

1. A scheme showing the general timeline for the experiments would be useful to better follow the manuscript.

2. In lines 215 and 218, the authors probably meant ”Fig. 1C” and not “Fig. 1D” and the other way around for line 221.

3. Channel rhodopsin AAV was injected in animals of 90 to 120 g of weight. Since there are reports of neuronal reorganization and changes in the PFC-vHC connection during an adolescent period (Thomases et al., 2014 JNS; Thomases et al., 2013 JNS), it would be important to discuss how this could affect the results and whether AAV expression could alter neuronal development.

4. Discussion ends abruptly and needs a final summary statement.

## Author Response

Dear Dr. Sisk,

Pleased find enclosed the revision of our manuscript titled “Plasticity of NMDA Receptors at Ventral Hippocampal Synapses in the Infralimbic Cortex Regulates Cued Fear”. We appreciate the thorough and thoughtful review. We have addressed all of the concerns and are submitting a significantly improved manuscript. Below we detailed how we addressed each concern.

Synthesis Statement for Author (Required):

Reviewers agreed that findings from these experiments are interesting and timely in investigating vHC-IL connections and synaptic plasticity in the context of fear conditioning and extinction. However, there were substantial concerns about some of the statistical analyses, interpretations, and conclusions. It is essential that you provide a rationale for the statistical analyses employed and report sample sizes for all groups and exact p values. Please also address each of the following points in a revised manuscript.

Major Concerns:

1. Full statistics would be more useful to understand the results. The number of subjects or cells should be stated for each experiment in the main text or in the figure captions. Exact p values and effect sizes should be reported.

Response: The number of subjects and cells for each experiment were added in the main text along with the exact p values and effect sizes.

2. The representative expression of the vector in Fig 1A shows AAV-mediated YFP expression in the hippocampus, however there is also considerable expression in cortex & amygdala transition area, which includes posterior basolateral amygdala, posterior piriform, and entorhinal cortex. The authors cannot rule out that the plasticity is primarily occurring at synapses from neurons from these areas projecting to the IL. This makes it difficult to support the authors' interpretation that vHPC-IL circuits specifically have the plasticity phenotype studied. Further experiments may be required to demonstrate whether these regions project to IL. Alternatively, authors need to adjust their interpretation to include other potential inputs to IL.

Response: The following sentences were added to the manuscript under Methods/ Stereotaxic Surgery:

After completing behavioral training, animals were euthanized for acute brain slice preparation. In addition, brain sections containing the hippocampus were fixed and prepared for further confirmation of EYFP expression in the ventral hippocampus.

Although we used established coordinates for infusion of the viral constructs into the rat ventral hippocampus (Sierra-Mercado et al., 2011) and we confirmed expression of viral proteins in the ventral hippocampus (Figure 1A), it is not possible to completely rule out the possibility that some fibers from viral expression in areas near the ventral hippocampus also contribute to the inputs recorded in IL. However, based on the relatively robust projections from the ventral hippocampus to IL (Hoover and Vertes, 2007), the majority of inputs activated in IL likely come from the ventral hippocampus.

3. The authors report correlations as an indicator of a relationship between the freezing behavior and NMDAR plasticity, therefore implying that the plasticity may underlie behavioral changes. However, the authors attempt to make conclusions from improperly performed correlations across all groups. The authors have already demonstrated that NMDAR currents are significantly different between groups, which matches the same pattern that they observe in the behavior, thus the correlation is likely due to group differences and not attributed to individual variation. In order to demonstrate individual variation, and thus a clear relationship between the behavior and plasticity, the authors need to conduct correlations within groups. A significant correlation within the COND group that is not present in the PSEUDO group and reversed in the SAME (and/or DIFF) groups would be required to make the conclusion that the plasticity and behavior are causally related.

Response: We agree that group differences can affect the correlations. However, if one variable does predict the other variable then it should be true for all groups. We do not believe that relationship should change depending on the treatment, unless other variables have been altered in one treatment that, thereby, alter the relationship between the 2 variables in the correlation. Since the correlations are weak at best, do not indicate causality and do not add significantly to our conclusions, we have removed the correlations from the results and text. Since fear conditioning and extinction are known to induce other synaptic and intrinsic changes, it is not surprising that the correlations between a single synaptic change and behavior are weak.

4. Discussion of how individual variation (“PTSD-like behavior”) in extinction predicts NMDAR plasticity is difficult to understand and may be a misrepresentation of the actual findings. The authors have not demonstrated that the individual variation predicts susceptibility to PTSD-like behavior (see #3 above) and need to avoid making broad assumptions about their current data. Discussion of how the individual variation in behavior, which predicts differences in NMDAR current & conductance, is similar to PTSD should therefore be relegated to the discussion only, and PTSD language should be removed from the results section.

Response: We have removed PTSD language from the results section.

5. The discussion of successful vs unsusccessful needs to be described in more detail in the Results section (and potentially Methods). Were the rats in the SAME context cohort in 2A treated with the same protocol as the rats in 4A, as the paper implies?

Response: Yes, the rats in the SAME group in 2A are the ones named SUCCESFUL in figure 4A. We further clarified this in the results.

If so, why were there no “unsuccessful” rats in 2A?

Response: The purpose of the experiments in figure 2A was to compare synaptic changes between animals that showed efficient learning and memory of the behavioral training that they were assigned (Figure 1D). Since some animals (6) from the SAME group did not extinguished fear they were excluded from the analysis shown in Figure 2 and 3. However, since electrophysiological recordings were done blindly in respect to the behavioral outcome, at the moment of analyzing data we noticed these differences on behavior. We further decided to compare electrophysiological recordings from neurons of the SAME cohort that showed SUCCESFUL extinction retention (7 rats / 23 neurons) with the ones that showed UNSUCCESSFUL extinction retention (6 rats/ 16 neurons).

What is the average spread of freezing across the same context cohort?

Response: To address this question two additional graphs were added to Figure 4, labeled as A2 and A3. These figures give more information about the distribution of freezing between the SUCCESFUL and UNSCUCCESFUL group, and show more clearly how they separate behaviorally.

Was the cohort in Fig 4A an anomaly?

Response: We think that it would not be completely accurate to call the cohort an anomaly. We do not know why some animals fail to show good extinction memory. However, we and others have reported that some animals show failed extinction (Burgos-Robles et al., 2007a; Peters et al., 2010; Cruz et al., 2015). In fact, one study examined about 50 male and 50 female rats and found that there was a wide spread of fear recall (Gruene et al., 2015).

The rats that show the PTSD-like poor extinction recall cannot be planned which makes them difficult to study. In fact, this makes them resemble PTSD in humans, which also occurs in a currently unpredictable and seemingly random fashion. Probably if we used very large groups like Gruene et al. 2015, we would always have some extinction failures; however, that is not cost or time effective. However, when they occur they make a very good group for comparisons.

We do not know why animals from the DIFF group cohort did not show unsuccessful extinction retention. This could be from the small sample size or because they were extinguished and tested in a novel context.

In the experiment shown in figure 4, the animals were divided into successful and unsuccessful extinction using a cutoff of 50% time freezing. However, this seems arbitrary. Is this always a population cut-off (pre-set value) or does this change based on the cohort? How is unsuccessful extinction defined? In many studies, animals show freezing responses as lower as 30%, and those are considered significant memory retention scores.

Response: To help to understand our rationale behind the behavioral cutoff, we added two additional graphs to Figure 4, labeled as A2 and A3, in which the behavioral differences are more evident. Also, our cutoff is based on a comparable cutoff that was previously reported (Burgos-Robles et al., 2007b; Peters et al., 2010).

6. One of the main conclusions from experiments depicted in figures 2 and 3 is that the AMPA/NMDA ratio is reversed after extinction. However, the data points at something perhaps more subtle that is yet to be identified. The concern comes from the results observed in the DIFF group, which extinguished at the same level than the SAME group, but does not show significant differences in AMPA/NMDA ratio or NMDA EPSC with respect to the COND group. However, these differences are also not significant compared to the SAME or PSEUDO groups. It would be useful to see the exact p values for all the comparisons and the effect sizes. Also, could the authors test the validity of the null hypothesis? We believe there could be more information to gather from these experiments, simply claiming, “extinction in a novel context failed to reverse the depressed NMDAR currents” is misleading, as the results do not seem to point in that direction.

Response: This paragraph was included in the results:

It is important to point out that the DIFF group received extinction and extinction recall in a novel context. Therefore, they did not extinguish their fear to the conditioning context. If the DIFF group had been placed in the conditioning context during recall they would likely have shown fear renewal and higher freezing than the SAME group.

We have also added the exact p values and the effect sizes.

7. The bar chart in figure 5A does not reflect what is stated in the main text. Again, we understand that the statistical analysis the authors did not detect a significant difference, but simply stating, “Although the contextual fear varied among the groups (Fig. 5A), there were no significant differences among them (F (3, 25) = 2.394, p = 0.092) does not reflect the nature of the results. In fact, the p value is close to 0.05 and we assume that effect sizes are large. This could be resolved by increasing the number of subjects or assays; however, there is no mention of the number of subjects or cells nearly anywhere in the manuscript.

Response: We added the sample sizes to the text. Effect sizes were calculated and added to the main text. As expected the effect sizes show that the COND and UNSUCCESSFUL groups showed more contextual fear than the PSEUDO and SAME groups. This also suggests that the failure of the DIFF group to extinguish the fear to context A likely also contributed to its failure to show significant reversal of the conditioning-induced changes in NMDA currents.

8. Although we understand the logic for doing experiment 7, we believe the comparison with the study done by Peters et al. is limited. The authors conclude, ”BDNF decreased the AMPA/NMDA ratio and increased the NMDAR currents in vHC-IL synapses mimicking the effects produced by fear extinction“. Since BDNF was applied to the bath, it is not possible to associate this mechanism with the one described in the previous experiments. A more comparable experiment would consist on BDNF injection into IL before extinction and slice preparation from these animals. In this case, they could compare memory retention scores with AMPA/NMDA ratios based on the effects of BDNF. In addition, it is unclear if the plasticity reversed by BDNF is acutely disrupted, leading to extinction effect. Additional experiments or appropriate justification may be needed to understand the molecular mechanism for BDNF-mediated changes in extinction and plasticity.

Response: We agree that we cannot say that the BDNF is responsible for the synaptic changes induced by extinction. However, we can say that incubation of slices with BDNF induces similar changes in NMDA currents as those found in animals after fear extinction. These effects are independent of any circuits that may be activated by an infusion of BDNF into an animal.

We think that the sentence mentioned above may have been unclear. We modified the sentence to say: ”BDNF decreased the AMPA/NMDA ratio and increased the NMDAR currents in vHC-IL synapses mimicking the synaptic effects produced by fear extinction“. We wish to indicate that incubating with BDNF produced similar synaptic changes as we saw after fear extinction. The strength of our experiments lies in the fact that multiple experiments support the conclusion that extinction increases the NMDA currents at vHPC to IL synapses. If we only had the data from the BDNF incubation, it would be difficult to know whether the effects reflected extinction-related changes. However, the fact that extinction produced similar changes as incubation with BDNF strongly suggests that these are extinction-related changes. Furthermore, animals that failed to recall extinction do not show these changes.

9. Discussion, lines 339-343: ”the fact that the group that received cued fear extinction in the novel context showed intermediary changes in the NMDAR EPSCs compared to the group that received extinction in the same context as fear conditioning suggests that extinction of fear to the cue and extinction of fear to the context both contribute to the synaptic modifications of the vHC-IL synapses.“ We wonder whether this can be concluded from these results or more experiments are required. There is an interesting correlation analysis done in figure 6, maybe it would be worth establishing whether AMPA/NMDA ratio correlates with freezing behavior in the DIFF group; this could give additional information to what is going on there.

Response: We agree that it is very difficult to disentangle the effects of contextual and cued fear extinction. We chose one method, which is to compare extinction in the same context to extinction in a novel context. The only differences between the two groups is the context. Therefore, it is likely that any differences in the synaptic changes are due to the difference in context. It certainly suggests this.

As indicated above the linear correlations between synaptic changes and freezing behavior are weak and do not suggest causality and have been removed.

10. In lines 369 to 373, the authors discussed their BDNF result. We found this discussion unsatisfactory for the reasons mentioned above. The authors could mention the limitations of this study in comparison with Peters et al. instead of just stating that BDNF may act through activation of Fyn kinase, which sounds a bit incomplete and random, as many other cascades could be involved. No discussion or justification for mention of Fyn kinase is given.

Response: We agree and have included discussion of the limitations of the comparison with Peters et al.

Minor points

1. A scheme showing the general timeline for the experiments would be useful to better follow the manuscript.

Response: A scheme of the general timeline for the experiments was added to Figure 1, labeled as letter E.

2. In lines 215 and 218, the authors probably meant ”Fig. 1C“ and not ”Fig. 1D” and the other way around for line 221.

Response: The error observed in the text in lines 215, 218 and 221 was corrected in the manuscript.

3. Channel rhodopsin AAV was injected in animals of 90 to 120 g of weight. Since there are reports of neuronal reorganization and changes in the PFC-vHC connection during an adolescent period (Thomases et al., 2014 JNS; Thomases et al., 2013 JNS), it would be important to discuss how this could affect the results and whether AAV expression could alter neuronal development.

Response: Although it is possible that AAV-mediated expression of proteins can alter cellular processes including synaptic connections, all groups would likely be affected equally. This is a general concern with all AAV expressed proteins and is not limited to adolescent expression. In the studies by Thomases et al. the changes in vHC induced plasticity in the mPFC was induced by systemic blockade of NMDAR during adolescence. It is not clear whether local expression of channelrhodpsin in the vHC during adolescence could produce similar changes.

4. Discussion ends abruptly and needs a final summary statement.

Response: We added a final summary.

We feel that we have adequately addressed the reviewers' concerns and have submitted a much improved manuscript suitable for publication in eNeuro.
